# An exploratory qualitative interview study on grassroots esports in sports clubs

**DOI:** 10.3389/fspor.2024.1405441

**Published:** 2024-08-01

**Authors:** André C. K. Baumann, Ståle Pallesen, Rune A. Mentzoni, Eirin Kolberg, Vegard Waagbø, Anders Sørensen, Joakim H. Kristensen

**Affiliations:** ^1^ Gamers Performance, Oslo, Norway; ^2^ Department of Psychosocial Science, University of Bergen, Bergen, Norway; ^3^ Norwegian Competence Center for Gambling and Gaming Research, University of Bergen, Bergen, Norway

**Keywords:** volunteers, esports, sports clubs, grassroots, community, leaders, competency, organization

## Abstract

**Aim:**

The current study aimed to explore grassroots esports in sports clubs in Norway from the perspective of volunteer esports leaders.

**Method and results:**

Fifteen volunteers were recruited from grassroots esports initiatives in various sports clubs and were interviewed via online video conferencing using a pre-developed semi-structured interview guide. Data was analyzed using inductive thematic analysis with a realist approach, which generated the following themes: (1) Local community impact at the center of motivation, (2) lack of support threatens the operations of the initiatives, and (3) competency development to overcome barriers. The participants perceived the grassroots esports initiatives as essential for children in the local community and as the core of their motivation as volunteers. Several challenges were mentioned for sustaining the initiatives, such as maintaining motivation, resource management, and recruiting new volunteers. Finally, competency and qualified esports trainers were mentioned as necessary for a high-quality offer.

**Conclusion:**

The grassroots esports initiatives in sports clubs are viewed by volunteer esports leaders to affect the local community positively. However, there are challenges tied to the operation of such initiatives, such as engaging volunteers and raising competence. Future research should investigate barriers to help develop strategies to support grassroots esports initiatives.

## 1 Introduction

Esports (electronic sports) is a casual or organized form of competitive gaming that has gained popularity in recent decades. Although esports is competitive, there are still discussions about whether it is a sport (1–4). In this context, grassroots esports represent gaming in an organized form and are primarily volunteer-driven and nonprofit-based akin to other sporting activities. Volunteers are an essential resource, and their contributions range from administrative tasks to social and physical support, ensuring that the organization's activities can continue (5). Four dimensions are essential in defining voluntary work: free choice, remuneration, structure, and intended beneficiaries (6). Voluntary work is the backbone of the grassroots sports clubs in Norway. At least 90 percent of all work done in the vast majority of grassroots sports clubs is done by volunteers, proposing that voluntary work is at the very foundation of these clubs (7), providing integral societal institutions where people meet, socialize, and create social capital (8). Moreover, the clubs are essential for improving sports performance and as a structured support system for the participants (9). While this has been the case for some time concerning traditional sports, esports, on the other hand, has recently emerged as an attractive sporting activity, but without the same supporting structures (10).

The grassroots esports initiatives target predominantly children and adolescents and have become an alternative to traditional sports in sports clubs (10). Trying to create a safe space and a sense of belonging, esports in sports clubs may have several positive impacts on the participants and the local community (11). The esports athletes typically gather on the sports club's premises and play in an inclusive environment with physical activities often organized in conjunction with esports sessions (12). While the most popular esports are not physically demanding compared to traditional sports like soccer or athletics, there is evidence that pain and injury occurrences within esports players are prevalent (13, 14). Including physical activity in grassroots esports initiatives might prove preventative for such negative outcomes, although the data on the frequency, type and duration of physical activity in grassroots initiatives have yet to be investigated.

Sports clubs' involvement in esports dates back to 2003, with SSV Lehnitz as a pioneering club. In 2015, the sports club Besiktas Istanbul established its esports department and is often considered the first recognized sports club to also have a renowned esports team (15). Over the years, high-profile clubs and a number of grassroots sports clubs have become involved in esports. By 2019, over 400 sports clubs and teams at varying levels worldwide offered esports as an activity (15). In Norway, esports in sports clubs have grown in popularity during the last few years, and an increasing number of sports clubs now accept esports as a legitimate activity in parallel to traditional sports (10). In January 2024, Norway's largest umbrella organization for volunteer grassroots esports initiatives had 115 unique member organizations, with 92 of them being sports clubs (16). However, this number accounts for approximately half of the esports initiatives in sports clubs in Norway (17). Based on data collected by the Norwegian Olympic and Paralympic Committee and Confederation of Sports during the spring of 2023 (17), it was reported that 179 of 9,454 sports clubs in Norway offered esports. Hence only a minority of sports clubs include esports. In comparison, the Football Association of Norway reported the number of sports clubs offering soccer to be 1,704 (18). Although esports lag behind the most popular grassroots sports, esports in sports clubs are expanding.

Grassroots esports in sports clubs seems to develop positively in Norway. Nevertheless, there might be potential challenges connected to the initiatives. With the organizational structure within sports clubs, volunteers play an essential role in establishing and operating grassroots sports (7). Common barriers hindering volunteerism include knowledge gaps, skillsets, or financial uncertainties (e.g., travel expenses and food) (19). There is currently no literature providing insights on this matter in grassroots esports. However, one can hypothesize that the abovementioned barriers also apply to esports.

Additionally, as esports is a relatively recent phenomenon in grassroots sports clubs, a framework for designing and running the initiatives might be beneficial. While traditional sports have an existing organizational structure within the sports clubs, esports need to find its place within these institutions. Common denominators between esports and traditional sports in how they are run are likely to exist, facilitating shared knowledge and resources (20). On the other hand, they may compete internally and externally for members, economy, and volunteers (20). While esports has had significant development in the last couple of decades, sustainability concerns exist, implying its ability to persist. Inclusivity, industry structure, and economy are all potential risk factors for esports' survival (21).

Few studies have explored the grassroots esports phenomenon in sports clubs. This study aims to explore the uniqueness of grassroots esports in sports clubs from the volunteer leaders' point of view. With this aim in mind, we desire to provide insights from the volunteer leaders on the operations, barriers, and development of the initiatives. Furthermore, by analyzing the insights provided by the participants, we can postulate practical implications for developing grassroots esports in sports clubs.

## 2 Method

The present study used a qualitative interview study to explore the grassroots esports phenomenon in sports clubs. The study was embedded in a realist epistemic approach. This approach implies that interviewees' accounts are understood as relatively accurate descriptions of their own experiences (22). Moreover, the study design and subsequent data analysis were based on an inductive approach (23), hence not being guided by any pre-existing theoretical perspective on grassroots esports. This approach was chosen due to the phenomenon explored in the current study, (i.e., grassroots esports in sports clubs) to our knowledge, have not yet been investigated in the esports literature.

### 2.1 Subjects

A tentative sample size estimation that assumed a population theme prevalence of 30% and a desired number of theme occurrences of 3, suggested that 14 participants were necessary to develop themes sufficiently (24). Fifteen esports leaders of grassroots esports initiatives were recruited through a digital recruitment poster, distributed online by the Grassroots Esports Alliance (25) and the Active Gamer network (26) on their social media channels. Both are established grassroots esports umbrella organizations in Norway.

The names of the participants were altered to ensure anonymity when presenting data extracts in the “Results”-section”. All participants signed an informed consent form before the interviews. The inclusion criterion was that the study participant had to be the current leader of the grassroots esports initiative in a sports club.

### 2.2 Procedure

The authors developed a semi-structured interview guide that included questions on a range of relevant topics, such as the study subjects' motivation for working with the esports initiatives, what defines a grassroots esports initiative, the role of physical activity, nutrition, and sleep for grassroots esports, and the framework for developing grassroots esports. The interview started with a short introduction describing the aim of the study and how data would be managed, followed by questions from the interview guide. The interviews were conducted through online video conferencing during March and April 2023. Interviews lasted between 45 and 75 min. Following the interviews, all participants received a gift card with a value of 500 NOK (∼ 45 EURO) as compensation for the time spent. Two authors (VW & AS) conducted the interviews and transcribed the audio recordings verbatim.

### 2.3 Analysis

Thematic analysis (TA) was used to analyze the transcribed interviews and develop themes across interviews (23). An inductive and semantic approach was employed in developing the themes, which implies concentrating on the explicit meanings of the data material (23). The first author (AB), who has competence and experience in working with and supporting grassroots esports initiatives, developed the coding and themes. The TA analysis followed the six-step process outlined by Braun and Clarke (23). The first step involved transcribing the interview data and generating ideas for the initial codes. In step 2, the initial codes were generated and highlighted in the transcripts using Quirkos (qualitative analysis software). The third step involved generating themes based on the collated overlapping codes and creating an initial thematic map. More specifically, themes did not “appear” but were rather developed through iterative processing of the data informed by the competence and experience of the first author (27). The fourth step involved rereading the transcripts and reviewing the existing themes, before collapsing the themes and generating a final thematic map. Step five and six involved defining and refining the themes and writing the report.

## 3 Results

Based on answers from 12 of the 15 participants, the average age of the interviewees was 41.5 years, ranging from 23 at the lowest to 55. Eight of the participants were women, while the remaining were men. Of all study participants, eight (two women) were gamers, meaning they reported playing computer or console games themselves regularly. The most frequently reported range of esports athletes per club was between 41 and 50, with the number of esports members ranging from zero (at start-up) to 80 per club. Information about the number of girls in the grassroots esports clubs was collected, with an average of 2.9 girls per club, ranging from zero to ten, representing a small minority of the player base. The primary user group was between 9 and 12 years old. Seven participants reported that the number of computers available at the sports club ranged from 10 to 25. The initiatives offered eleven different esports in total, with the three most popular being Overwatch 2, Rocket League, and Fortnite. Eight participants reported that each training session lasted about 120 min. Of all participants, seven reported having two weekly esports training sessions. Finally, the participants reported what they believed to be essential for the grassroots esports initiative. Although the response varied between the participants, the following topics were mentioned: facilities, esports trainers, competency, grassroots leagues, recruiting volunteers and parents, structured esports training, sufficient economy, cooperation with the sports club, fair play, a code of conduct, and the importance of having a framework for developing the grassroots esports initiative. The three overarching themes structuring the remainder of this section are:

1. Local community impact at the center of the motivation
2. Lack of support threatens the operations of the initiatives
3. Competency development to overcome barriers

### 3.1 Theme 1: local community impact at the center of motivation

This theme relates to the participants’ descriptions of the reasoning behind the involvement in the esports initiative. Most participants' motivation to work with the esports initiatives originated from a sense of positive impact on the local community. Almost all participants reported that their grassroots esports initiative had a positive impact on children in the local community and that it provided them with a safe physical location for socializing:

"The positive thing is that we have an offer for them. Before, they have just stayed home, maybe been bullied and teased and kept out, or felt that they were different in other ways, but now have a place where they feel they can come [and meet others].” (Brenda).

About half of the participants stated that the esports initiative's effect on the younger children was paramount for the children and the local community, and most of them mentioned that creating a social hub where children could meet and play together was their most important mission. Examples of this were meeting the children at their arena of interest, using club premises in the local community for the esports initiative, and that it was highly socializing for the children:

"The initiative is for the children in primary school in particular. When they are going back to school on Monday, they can say that they have also done something during the weekend. It hurts to meet those who have nothing. This [the grassroots esports in the local sports club] is all they have. You meet all levels of society, but when we open the doors and it’s free, people come and they're very grateful. It means so much when you create a sense of belonging in the local community.” (Tom).

Several participants reported that grassroots esports initiatives engage children who are perceived as not fitting into the local community. Thus, the children are provided an arena to find new friends with common interests. One participant shared an example of the initiative's inclusive effect on children not participating in typical sports arenas:

"(…) Yes, I have given them the opportunity through grassroots esports to include individuals who may not have so many other social arenas. To help them participate in grassroots esports, and I have done that through contact with the different parents where they [the children] have tried football and handball, and all the other physical sports without much success. (…)” (Stuart).

Some participants provided examples of different positive social effects on the youth in the local community, such as social networking outside of the esports premises and forming of new friendships. One participant also mentioned that a few children had begun attending school again after participating regularly in the esports initiative. These positive social effects, in turn, provided gratification to the esports leaders:

"Yes, another thing is that we know that across different esports groups [in the sports club], they have started to meet outside of training. They have formed friendships and suddenly met at each other’s houses. They did not know each other before. That is very gratifying.” (Alice).

Several esports leaders mentioned that the initial motivation for establishing the initiative was for their children. A few also reported that watching the children having fun and developing their identity was engaging and motivating. However, it seemed like the core motivation to continue working in the esports initiatives was related to the impact on the local community and other children:

"(…) But I have continued because I see how important it [the esports initiative] is. Many parents have come and said that if it had not been for this, my son would have just stayed inside.” (Jill).

"(…) As I said before, esports has always been about her [Marýs daughter] being a participant in it. However, when I got to know other children, got to know other parents, and I realized how much importance it has for a lot of different young people out there —having an offer of this dimension that perhaps not so many others have has been the driving force” (Mary).

### 3.2 Theme 2: lack of support threatens the operations of the initiatives

The second theme describes the participants' perceptions of a large workload and lack of understanding and support. Although many sports clubs now offer grassroots esports, they are reliant on volunteers. Most participants indicated a lack of general support for esports initiatives. Some participants hypothesized that the lack of understanding and support originates from prejudices towards esports initiatives. Another challenge was to engage parents to enlist as volunteers.

Most participants described that one or a few key individuals were the impetus for establishing and operating the initiative. Different assignments, ranging from administrative tasks to operating physical and esports training, strained single individuals, resulting in a significant workload. Several participants suggested that more volunteers were needed in the esports initiative to help them organize the activities. One participant explained that there had not been many challenges, although he could have used for more individuals with the same capacity as himself:

"So, I would not say that there have been so many big challenges, other than that there should have been more hours in the day, and I should have had four copies of myself.” (Brandon).

To combat resource management issues, the esports leaders reported that including and recruiting parents is essential to help sustain the esports initiative. However, the recruitment process for several clubs was challenging when the parents perceived esports as too difficult to coordinate at the grassroots level. A few participants questioned why other parents could not understand that esports-specific competence is not a prerequisite for administrating esports training. Most of the participants argued that they perceived the parents as unwilling to volunteer due to their lack of knowledge of esports, both practically and theoretically:

"(…) In the sports club itself, it has been difficult, I would say, because you have been alone, and then there are not that many others to engage [to get help from]. It is not easy to get parents involved because they have not played esports. They think that they can't contribute because they don't know the sport [esports], so we've tried to get them involved with the fact that they can be coordinators, to check that everyone is doing ok, and that there’s a lot you can do which doesn't have to do with the esports directly.” (Jill).

Most participants voiced that the parents are unaware of how they operate and what the children are doing in the grassroots esports initiatives and that this unawareness and lack of support are concerning:

"What I see as the biggest challenge is that parents don't understand. We say that we have physical activity in conjunction with the esports training, and subsequently we get questions from the parents that think that we are teaching their kids in another sport [with esports being “the sport”], when we just offer a variety of different physical activities” (Carmen).

“Yes, there is far too little interest. That is, it is usually the coach plus the players. The parents do not even bother to watch and see how good they are.” (Johnny).

A few participants noted that the parents were unaware of how they operated because they believed the parents had not played esports and thus were unfamiliar with the activity. One mentioned that a potential cause for the lack of involvement, and perhaps lack of respect for the initiative, is due to prejudices:

"We can meet a bit of prejudice with that they [the grassroots esports participants] get even more screentime. After all, we are still at that point where some parents may be negative [to additional screen time], so they don't take it seriously. Maybe that’s why they don't pay the membership fee.” (Betty).

A common difficulty for the volunteers was getting acknowledgment for grassroots esports and explaining its operation and vision, that the initiative is something other than a gaming concept. The resistance appears on different levels, from the lack of understanding and difficulty obtaining the necessary infrastructure:

"But it is an exercise in, what can I say, being punched in the face repeatedly and continuing, because you meet resistance on so many levels. You struggle to get hold of premises, and you struggle to get hold of suitable trainers. You constantly have to explain that you are not a gaming initiative [but an organized esports initiative]. You encounter very little understanding of what you do.” (Tom).

### 3.3 Theme 3: competency development to overcome barriers

The third and final theme relates to the participants' perceived need to develop competencies to improve the esports initiatives. Most participants reported multiple barriers to establishing and operating grassroots esports initiatives. One of the most common barriers mentioned was the need for trainers with esports-specific competence. Lack of such competence made the training sessions demotivational for some children. Several participants also mentioned the need for additional support for the esports initiatives through an association. Finally, the need for specific guidelines in the esports initiatives was mentioned by several participants, who needed help communicating and establishing guidelines for health and performance, suggesting that the parents were essential in this process.

Several participants mentioned the need to increase the esports-specific competency of the parental esports trainers as they often lack sufficient esports-specific competence to enhance the esports training:

“(…) it’s also a huge challenge [about not having paid coaches] because then there will be parents who have no gaming experience who just let them play, and then they learn absolutely nothing”. (Brenda).

One participant emphasized the challenge of obtaining enough esports competence to the club, proposing a solution to apply for funding to hire experienced esports coaches:

"What I could call a dream scenario for me would have been that you could apply for money somewhere, to get a salary for esports coaches for a while, to bring in that kind of expertise [esports-specific competence].” (Jill).

A challenge connected to the lack of esports competence of the esports coaches was viewed to arise when the athletes themselves were more proficient and knowledgeable, albeit the reason for this was not disclaimed during the interviews:

“(…) And then it becomes difficult when the athlete is much better than the coach and has much higher competence than what the coach has. And that has been difficult.” (Richard).

There were also comments about the need for esports-specific competence and the motivation to connect with larger esports organizations. The reason for connecting with such organizations was to interact with other esports initiatives and build a more professionalized structure for esports. Furthermore, a few participants mentioned that this connection could directly benefit the esports initiative in the sports club, although what type of benefits this entailed was not specified:

"Then you need people who can run it [grassroots esports initiatives], preferably with esports expertise. And then I'm wondering, we have not applied to any esports association, but it might be smart to join. Not only smart, but it’s probably also necessary to join one of these associations to get some help and support from it.” (Bill).

Expanding upon the lack of competence, most participants acknowledged the importance of good nutrition, enough sleep, and regular physical activity for health and esports performance. However, concrete guidelines or specific rules were not mentioned, and some participants stated that such guidelines are up to the parents of the participants, except one participant who mentioned that it was not allowed to bring sodas or energy drinks to the esports training.

"(…) No, we had a lecture [about health and lifestyle] for the parents last year. I think it is really the parents that we need to talk to more.” (Shannon).

"I come from a physical sports background, so I think these factors are essential [diet, sleep, and physical activity], and we talk a lot about it. We have even written a sports plan that includes these factors. (…) We talk about these factors during training but not systematically. A fourth grader [10 years old] does not decide when to go to bed themselves, you know?” (Carmen).

In contrast to the abovementioned responses, one participant felt ambivalent about communicating lifestyle guidelines to their esports players and felt inclusivity to be more important than demanding health and performance guidelines, especially for the players not competing at a high level:

"(…) But it’s [guidelines for nutrition, sleep, and physical activity] something that we sort of mention now and then that this is how we want it to be, but we don't feel that we are in a position to demand so much more now than maybe from our FIFA boys since they are playing at a high level.” (Betty).

## 4 Discussion

**Figure 1:**
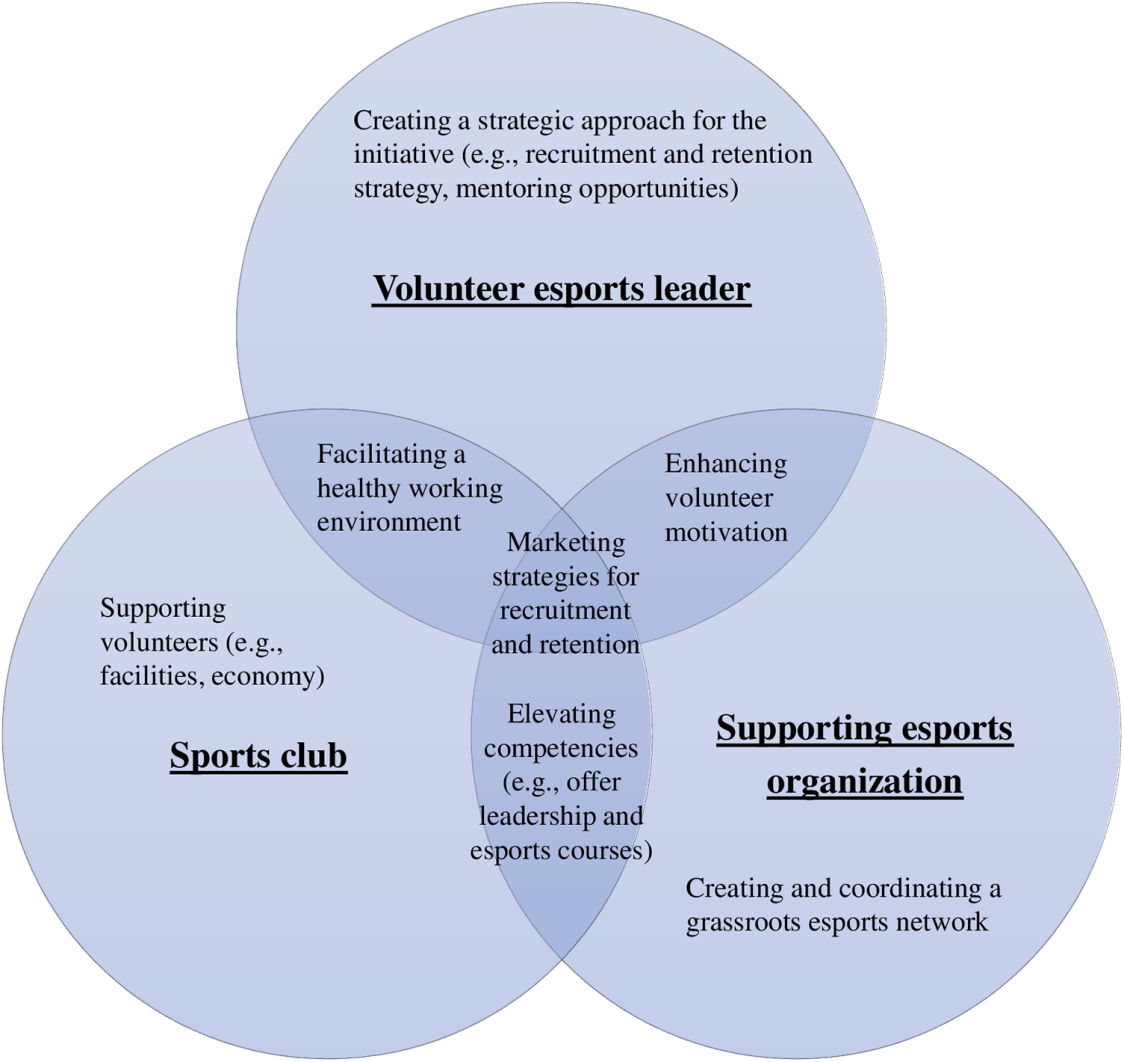
Venn diagram of potential key factors for success of voluntary grassroots esports initiatives.

The current study explored the grassroots esports initiatives in sports clubs from the perspective of volunteer leaders. Based on the present findings, the Venn diagram in Figure 1 shows potential key aspects that might impact a successful initiative. The diagram represents different factors the three stakeholders (sports club, esports volunteer leader, and supporting esports organization) can utilize to strengthen the initiatives, with the overlapping factors being potential collective efforts. Our discoveries have practical implications for supporting grassroots esports initiatives in sports clubs. Based on Figure 1, the following discussion emphasizes volunteer recruitment and retention through knowledge, marketing strategies, and organizational support.

### 4.1 Different motivations for volunteer engagement in esports initiatives

The first theme suggested that the volunteers' motivation for engaging with grassroots esports initiatives is rooted in positively impacting the local community and creating an environment with a bottom-up approach to grassroots esports. These motivational factors align with two other grassroots esports studies in Sweden (28, 29). In our study, eight of the esports' leaders reported themselves to be gamers of which six were men. A plausible explanation for the gender differences might lie in the motivation of volunteerism. The male leaders seemed more oriented toward the gaming activity itself. In contrast, this seemed less relevant for female leaders, as most of them disclosed their initial motivation to be their own offspring's involvement in esports or developing this arena for children in the local community. This resonates with sociological literature which indicates that women are more likely to volunteer in an unselfish, helping, and empathetic way compared to males (30).

### 4.2 Recruiting and retaining volunteers

Most participants in the current study painted a picture of being alone with much work and responsibility for the initiative. The main challenge was to engage and recruit parents of the children in the initiatives as esports coaches or volunteers. A traditional way of recruiting to sports organizations is through program participants, engaging the parents as volunteers (31). A fitting method of recruiting volunteers is essential to discover the right profile, focusing on using fewer recruitment methods and instead on quality over quantity (32).

Clary et al. (33) suggest that one should not make assumptions about the volunteers' motivation but rather assess it. By following this approach, recruitment strategies for finding the proper volunteers may be enhanced. If the goal is to draft volunteers familiar with gaming and esports culture, innovative methods such as digital marketing might be relevant recruitment strategies for grassroots esports initiatives (34). Wording can be crucial to avoid being unappealing in recruitment marketing, suggesting that familiarizing with the audience is imperative (35). As the average age of the volunteers in the current study was 41.5 years, some of these participants are likely to be parents of primary school children. Web-based strategies might be a viable solution to recruit this population due to their relatively low cost and alignment with the challenge of volunteer time management (35). However, Morrow-Howell (36) proposes approaching volunteers in person, which seems to be the best recruiting method. A strategy could be to host an open event for children and parents, which would lead to physical meetings with potential new volunteers.

If the initiatives have a high turnover of volunteer resources, qualifying new ones to replace those who leave can be expensive (37). Frequent replacements of volunteers can be a considerable challenge for the nonprofit initiatives (38), amplifying the role of a strategic approach such as good recruitment practices, an explicit retention strategy, as well as and mentoring opportunities (39). Furthermore, understanding the work culture is imperative for the initiatives' success (40).

### 4.3 Supporting the volunteers’ dedication through a robust organization

The grassroots esports supporting structures, such as sports clubs, umbrella organizations, or associations, are crucial for supporting the volunteers. Supportive organizational structures [e.g., network administrative organizations (NAOs) or umbrella organizations] can assist with initial motivation and retention of volunteer involvement (41). The NAO represents a separate entity that governs the network and its activities, functioning as an externally governed network broker, usually as a nonprofit or government entity (42). Peng et al. (43) discuss that creating the NAO can be crucial in sustaining the esports network due to the variety of competencies needed. The NAO must have high internal and external credibility to succeed.

Based on Provan and Kenis's definition, some esports umbrella organizations in Norway operate similarly to NAOs (42). The convergence of such organizations is crucial for the overall sustainability of the esports communities, highlighting the importance of collective action (44). Now, there seems to be a “substitute mode” for the esports associations in different countries, defined by a contest between associations on who should be the leading NAO (45). One should be concerned about the possibility of competing organizations in esports, which might hurt the development of the sport (44). As Witkowski (45) explains, the emergence of additional organizations competing for the peak position might impede the development of esports locally. However, the emergence of such organizations might prove inevitable if the current organizational situation is sub-optimal, for example in terms of a history of political scandals, employees, or board members with a lack of integrity (e.g., not working for the best of the esports as a whole), or the lack of support from the esports community. Therefore, deploying diplomatic strategies seems essential for sustainable development for the organization of esports in a way that is facilitative to the whole of esports (46). If one can create a well-functioning NAO, it might facilitate a unified approach with collaborative strategies for all parties involved. The NAO can be considered akin to what Murray et al. (46) propose through the esports diplomacy theory: “The theory is characterized by non-hierarchical, interdependent, and relatively stable relationships, embracing a variety of actors who share common goals and who exchange resources in pursuit of these goals.”

These supporting organizations possess tools to empower esports volunteers, such as promoting activities to make the volunteers feel part of a family. Activating the volunteers through these organizations is essential to strengthening the bond between the volunteers and enabling volunteer retention (47). Personal development, displaying competence, participating in a community, and promoting well-being are likely to promote volunteer retention if the volunteer assignments are congruent with their values (48). The success of NAOs or umbrella organizations supporting nonprofit grassroots esports clubs depends on members sharing the same beliefs and values (35). A recent study showed that esports organizations' possible strategies are building a supportive environment for volunteers through increased competency, trust, and transparency (49). Furthermore, the perceived organizational support will increase when the volunteers experience intrinsic (e.g., seeing youths grow and develop) and extrinsic (e.g., salary) rewards. Usadolo et al. (50) explain that organizations can support volunteer motivation with the abovementioned factors. Finally, the leaders of umbrella organizations and esports initiatives must understand the organizational needs to develop a good culture (51).

### 4.4 The lack of competency as a barrier to development

The participants perceived recruiting coaches with sufficient esports-specific competence as a challenge. Moreover, competence was lacking in nutrition, sleep, and physical activity, essential to promote healthy lifestyle habits.

A cross-sectional study from Soffner et al. (52) on the gaming and esports population shows that the fruit and vegetable intake is too low compared to the recommended intake. At the same time, energy drinks positively correlate with gaming and screen time (52). In terms of sleep routines, the literature on esports players reports conflicting results. A few studies report adequate sleep duration (53, 54), while others do not (55, 56). A recent study reported that esports coaches have suboptimal sleep hygiene knowledge, suggesting that esports coaches need more competence on how to monitor and advise their players for optimal sleep routines (57).

Some information and recommendations are available concerning the esports leaders' apparent need for more knowledge on specific lifestyle-related topics for esports athletes. A study by Baumann et al. (55) on esports students at colleges and folk high schools in Norway presents recommendations for tackling lifestyle-related challenges, for example, avoiding large meals before a competition, eating breakfast, having a suitable sleeping environment, avoiding blue-light-emitting devices before bedtime, and engaging in regular physical activity. Other potential strategies for improved health and performance for esports athletes can be obtained by having a nutrient-dense diet, avoiding alcohol, having a regular meal pattern, maintaining a healthy body composition, and being wary of caffeine intake (58). Pizzo et al. (59) report that traditional athlete performance techniques (e.g., healthy pre-game meals and warm-up exercises) have a contagious effect on the esports athletes in a sports club. This synergy where traditional sports can offer a developed framework for general lifestyle- and performance strategies to esports athletes seems like a low-hanging fruit to be considered.

Finally, sports coaching might have several benefits for esports youths, such as improved performance and development in terms of habits and structure, and can as such be a helpful tool for leaders and coaches in these grassroots initiatives (60). Nyström et al. (21) emphasize the structuring of the coaching process to develop competencies and communicate good practices, thus leading to a more sustainable esports development. A study on League of Legends head coaches by Watson et al. (61) revealed the need for consistently developing coaching skills due to the frequent changes of the sport (i.e., patches, expansions). In summary, developing appropriate education and recruitment schemes is essential for athletes as well as coaches (21). A valuable recruiting area in this regard might be schools that are offering an esports curriculum, where one might anticipate a larger group of the students being familiar with esports. However, few schools offer esports coaching as a part of their program and instead focus on esports businesses (62). Emphasizing coaching in the esports curriculum in the educational field might help recruit esports trainers to the grassroots esports initiatives and improve the trainers` competence.

## 5 Strengths and limitations

The current study is the first to explore the phenomena of grassroots esports in sports clubs from the volunteer leaders' perspective. A limitation of the study might be undiscovered themes during the data analysis process. It is likely that only participants already following the grassroots umbrella organizations' social media platforms would get notified about the study, potentially leading to a selection bias. Validation of the study results with a larger sample size as well as cross-culturally is needed. Although there is a clear male preponderance in terms of esports club membership, the sample of esports leaders had a relative even gender distribution. Overall, the proportion of female esports leaders in Norway is about 25%. This may imply that there was a certain gender-based selection bias to the present study. Still, the male preponderance in terms of membership far outweighs the male majority at the leadership level. Long-term, the relatively good representation of female esports leaders may have positive effects regarding recruitment of female esports members.

## 6 Conclusion

The current study explored Norwegian grassroots esports in sports clubs from the volunteer leaders' perspective and identified several critical issues for these initiatives. Findings generated from interviews with fifteen esports leaders show that the initiatives are typically volunteer-driven and dependent on the motivation of the individual leader. Secondly, significant barriers exist in terms of recruiting and retaining volunteers, primarily due to considerable workloads and a lack of understanding and support for the initiative. Finally, esports-specific and non-esports-specific competence regarding nutrition, sleep, and physical activity are needed. To help strengthen the initiatives, we propose a model involving cooperation between sports clubs, volunteer leaders, and supporting esports organizations. Based on the findings in the current study, the leaders, clubs, and organizations should focus on a systematic approach for recruitment and retaining volunteers by creating a clear and visible marketing profile and empowering volunteer collaboration, increasing the competency of the volunteer leaders and coaches through courses and formal education, and support through esports organizations. Future research should investigate the current study's findings in detail to explore how to develop the critical areas further to facilitate grassroots esports initiatives, with the core concern being recruitment and retention of volunteers.

## Data Availability

The raw data supporting the conclusions of this article will be made available by the authors, without undue reservation.
